# Trends and Associations of Physical Fitness and Dietary Behaviors Among Children Aged 10–12 Years in Ningxia Between 2019 and 2024

**DOI:** 10.3390/nu18152510

**Published:** 2026-08-03

**Authors:** Junying Liu, Xinxin Zhang

**Affiliations:** 1 Faculty of Education, Shaanxi Normal University, Xi’an 710119, China; nuoyuhan@snnu.edu.cn; 2 School of Physical Education, Shaanxi Normal University, Xi’an 710119, China

**Keywords:** physical fitness, dietary intake, school-aged children, China

## Abstract

Background: This study aimed to examine trends in physical fitness among 10–12-year-old children in Ningxia, China, from 2019 to 2024 and to investigate associations between dietary behaviors and physical fitness outcomes using repeated cross-sectional data from the National Student Physical Fitness and Health Survey. Methods: This repeated cross-sectional study included 3970 children aged 10–12 years from Ningxia, China (1960 boys and 2010 girls), based on data from the National Student Physical Fitness and Health Survey conducted in 2019 and 2024. Physical fitness indicators and dietary behaviors (breakfast consumption, egg intake, milk intake, and sugar-sweetened beverage consumption) were assessed using standardized assessments and questionnaires. Sex-stratified *t*-tests and multivariable linear regression models were performed to examine temporal changes and associations between dietary behaviors and physical fitness outcomes, adjusting for age, BMI, survey year, physical activity, and sleep duration. Results: From 2019 to 2024, both boys and girls showed improvements in multiple physical fitness indicators, including lung capacity, muscular endurance, and speed–agility performance, accompanied by increases in BMI. Adjusted analyses showed modest associations between dietary behaviors and specific fitness outcomes. Egg and breakfast consumption were associated with some measures of muscular endurance, whereas sugary beverage consumption showed associations with several fitness indicators, particularly among girls. Conclusion: Children in Ningxia experienced improvements in physical fitness from 2019 to 2024, alongside changes in body mass status. The observed associations between dietary behaviors and fitness outcomes highlight the potential relevance of modifiable lifestyle factors during late childhood. Integrated school-based strategies combining physical activity promotion, healthy dietary behaviors, and weight monitoring may support healthy development in this population.

## 1. Introduction

Children aged 10–12 years undergo rapid physical growth while also facing increasing academic demands, making this a particularly sensitive period for establishing healthy behaviors [1,2]. Physical health in late childhood plays a crucial role in overall development and well-being, as well as in the prevention of chronic diseases later in life [1,3]. In recent decades, declining physical activity levels and increasingly sedentary lifestyles worldwide have raised concerns about reduced physical fitness and rising rates of childhood overweight and obesity [4,5]. Globally, declining trends in several components of children’s physical fitness, including muscular strength, endurance and cardiorespiratory fitness, have been reported [6], yet some disparities exist between Asian and Western populations. Western children are increasingly affected by diets high in ultra-processed foods and sugar-sweetened beverages, while some Asian populations face a double burden of malnutrition, with undernutrition and overweight coexisting during nutrition transition [7]. These differences reflect distinct nutritional transitions and lifestyle changes across regions. These trends can negatively affect cardiovascular health, musculoskeletal development, and long-term health [2].

In China, improving the physical health of school-aged children has become a national priority [8], with policies emphasizing regular physical activity and healthy living. Recent educational reforms, such as the “Double Reduction” policy fully implemented in 2021, further aim to reduce excessive academic pressure, cut after-school tutoring hours, and guarantee sufficient daily physical activity at school to influence children’s daily routines and lifestyle behaviors [9]. However, disparities in fitness and lifestyle behaviors persist across regions—especially in less developed western China [10], and up-to-date evidence on recent physical fitness trends among children in these areas remains limited. Ningxia, a western region of China with diverse socioeconomic characteristics, ongoing nutritional transition, and marked urban–rural disparities, provides a unique context for examining how lifestyle changes influence childhood health [11,12]. Such characteristics make Ningxia a valuable setting for investigating variations in childhood physical fitness and dietary behaviors during regional socioeconomic and nutritional transitions.

Recent years have represented a period of substantial social and public health transitions in China, characterized by the COVID-19 pandemic and major educational reforms. The year 2019 provided a pre-pandemic reference point, whereas 2020–2022 reflected a period of disrupted lifestyles and reduced opportunities for physical activity among children [13,14]. The subsequent years represented a phase of social normalization and educational reform. This timeframe enables the evaluation of temporal trends in physical fitness and dietary behaviors among children in western China.

Dietary behaviors are a key modifiable determinant of childhood physical fitness and health [15]. Research has found that positive habits, such as regularly eating breakfast and consuming adequate amounts of protein and dairy products, are associated with better growth and development, muscle strength, and cardiorespiratory fitness [16,17,18,19]. In contrast, frequent consumption of sugar-sweetened beverages is associated with adverse health outcomes [20,21]. Currently, most research focuses on adolescents, with few studies examining the physical fitness status of children aged 10–12 and its association with dietary patterns, particularly in Western regions. Furthermore, much of the existing evidence has been derived from urban or economically developed populations, whereas evidence from multi-ethnic and rapidly transitioning Asian regions remains limited, potentially limiting the applicability of existing evidence to diverse child populations.

Therefore, this study aimed to examine trends in physical fitness indicators from 2019 to 2024 and to explore their associations with dietary behaviors among children aged 10–12 years in Ningxia, China. This study provides updated evidence on temporal changes in physical fitness and its behavioral correlates during late childhood. The findings will not only inform localized public health strategies for children in Ningxia and similar regions undergoing socioeconomic and nutritional transitions in western China, but also provide valuable references for other low- and middle-income multi-ethnic regions in Asia.

## 2. Methods

### 2.1. Participants

**Figure 1:**
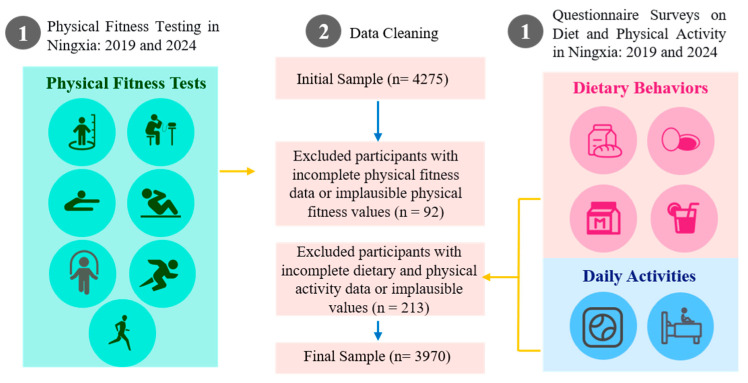
Flowchart of the study design and participants’ selection process.

This analysis is based on data from the Chinese National Survey on Students’ Constitution and Health (CNSSCH) [22,23,24], the most comprehensive nationally representative survey of student health indicators in China. This study employed a repeated cross-sectional design. Data were collected from children aged 10–12 years in Ningxia during 2019 and 2024. Participants from the two survey periods were independent samples rather than the same individuals followed longitudinally. The schools and classes sampled in 2019 and 2024 were also independent, and no schools, classes, or students were followed longitudinally across survey years. The initial combined sample from 2019 and 2024 included 4275 participants. During data cleaning, participants were excluded according to predefined criteria. First, 92 participants were excluded due to incomplete physical fitness data or implausible physical fitness values identified during quality control procedures. Second, an additional 213 participants were excluded due to incomplete dietary and physical activity questionnaire information or implausible values in these variables. After these exclusions, the final analytical sample consisted of 3970 children (Figure 1). The study protocol was approved by the Ethics Committee of Shaanxi Normal University (201916001, 2019-09), and written informed consent was obtained from all participants and their legal guardians.

### 2.2. Data and Measurement

#### 2.2.1. Physical Fitness Assessment

Physical fitness was assessed according to the standardized protocols of China’s National Student Physical Fitness and Health Survey [25]. The evaluation encompassed anthropometric measurements, including height and weight, from which body mass index (BMI) was calculated as weight (kg) divided by height squared (m^2^), as well as a series of physical performance tests. BMI was included as an anthropometric indicator to describe changes in body size and weight status across survey years. BMI categories (normal weight, overweight, and obesity) were classified according to age- and sex-specific reference criteria [26,27]. Under standardized conditions and following a standardized warm-up, trained physical education teachers administered tests of cardiorespiratory endurance (50 × 8 m shuttle run), muscular strength/endurance (sit-ups), flexibility (sit-and-reach), speed (50 m sprint), and coordination (1 min rope skipping) (Table 1).

#### 2.2.2. Dietary Assessment

The dietary assessment tool was adapted from the standardized instrument used in the 2014 National Survey on the Physical Health Status of Chinese Students [23]. This instrument was developed for large-scale epidemiological surveys and designed to ensure feasibility and consistent implementation across diverse regions, including remote and resource-limited settings. Its simplified format reduces respondent burden and facilitates standardized data collection among school-aged children. Participants completed the questionnaire independently following a standardized protocol, with trained teachers providing guidance for primary school students when necessary. The dietary module included four questions assessing selected dietary behaviors, including breakfast frequency, egg consumption, milk consumption, and sugar-sweetened beverage intake [11,28,29,30,31,32]. Previous studies using this instrument have demonstrated its feasibility for assessing dietary behaviors among Chinese children and adolescents.

Diet 1: In the past 7 days, how many days did you eat breakfast?

Diet 2: In the past 7 days, how many days did you eat at least one egg?

Diet 3: In the past 7 days, how many days did you drink at least one glass of milk/yoghurt or soy milk?

Diet 4: In the past 30 days, how many times per day did you usually drink sugared beverages, such as cola, tea drinks, drinks with fruit juice, etc.?

For questions 1–3, participants indicated their dietary frequency on a scale of 0 to 7 days per week. Question 4 offered seven qualitative intake categories, ranging from “none” to “five or more times per day” (including “less than once per day”, “once per day”, “three times per day”, and “four times per day”) [23]. Physical activity levels were assessed using a previously validated questionnaire. Moderate-to-vigorous physical activity (MVPA) was derived from participants’ responses to two core items on the weekly frequency and average daily duration (in minutes) of light-, moderate-, and vigorous-intensity physical activity over the past seven days [33].

### 2.3. Statistical Analysis

All statistical analyses were performed using R software (version 4.4.3; R Foundation for Statistical Computing, Vienna, Austria). The 2019 and 2024 datasets were merged and analyzed separately for boys and girls. Continuous variables are presented as mean ± standard deviation (SD), and categorical variables are presented as frequencies and percentages.

For each sex, differences in anthropometric characteristics and physical fitness indicators between 2019 and 2024 were examined using independent-samples *t*-tests. Effect sizes for between-year differences were calculated using Cohen’s d. False discovery rate (FDR) correction based on the Benjamini–Hochberg procedure was applied to dietary–fitness association analyses and between-year comparisons of fitness indicators to account for multiple comparisons. Differences in BMI category distributions (normal weight, overweight, and obesity) between survey years were assessed using chi-square tests separately for boys and girls.

To investigate the associations between dietary behaviors and physical fitness outcomes, multivariable linear regression models were constructed separately for boys and girls. Breakfast consumption, egg intake, and milk consumption were included as independent variables. These dietary variables were treated as ordinal frequency variables according to their original questionnaire coding, with higher values indicating more frequent consumption. All models were adjusted for survey year, age, BMI, physical activity, and sleep duration. Standardized regression coefficients (β), *p* values, and FDR-adjusted *p* values were reported. Multicollinearity was assessed using variance inflation factors (VIFs), and all VIF values were below 2.0, indicating no evidence of problematic multicollinearity.

To further examine the overall associations between categories of sugary beverage consumption and physical fitness outcomes, Type III analysis of variance (ANOVA) was performed after adjustment for survey year, age, BMI, physical activity, sleep duration, breakfast consumption, egg intake, and milk intake. A two-sided *p* value <0.05 was considered statistically significant.

## 3. Results

### 3.1. Participant Characteristics

The final sample comprised 3970 children aged 10–12 years: 1447 (36.4%) enrolled in 2019 (722 boys, 725 girls) and 2523 (63.6%) in 2024 (1238 boys, 1285 girls). Gender distribution was balanced across both survey years (49.4% boys, 50.6% girls overall).

### 3.2. Trends in Physical Fitness Indicators Among 10–12-Year-Old Students from 2019 to 2024

**Figure 2:**
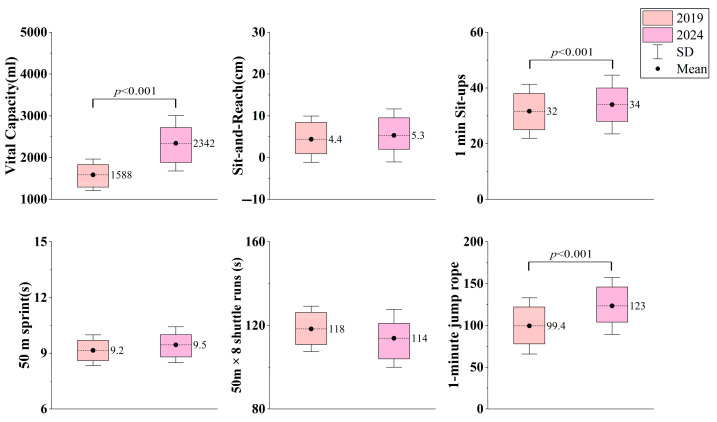
Comparison of physical fitness among boys in 2019 and 2024.

Among primary school boys, BMI increased significantly from 2019 to 2024 (Table 2). Regarding physical fitness performance, vital capacity showed a marked improvement from 2019 to 2024 (t = −27.99, *p* < 0.001, Cohen’s d = 1.31). Sit-up performance also increased significantly, indicating improved muscular endurance (t = −5.01, *p* < 0.001, d = 0.23). In addition, the completion time of the 50 m × 8 shuttle run decreased significantly in 2024, reflecting increased speed–agility endurance performance (t = 7.50, *p* < 0.001, d = 0.35). Jump rope performance increased substantially over time (t = −15.09, *p* < 0.001, d = 0.71). Flexibility, assessed by the sit-and-reach test, also increased significantly, although with a small effect size (t = −3.23, *p* = 0.001, d = 0.15). In contrast, 50 m sprint performance did not improve, with a significant increase in completion time observed among boys (t = −6.91, *p* < 0.001, d = 0.32) (Figure 2).

**Figure 3:**
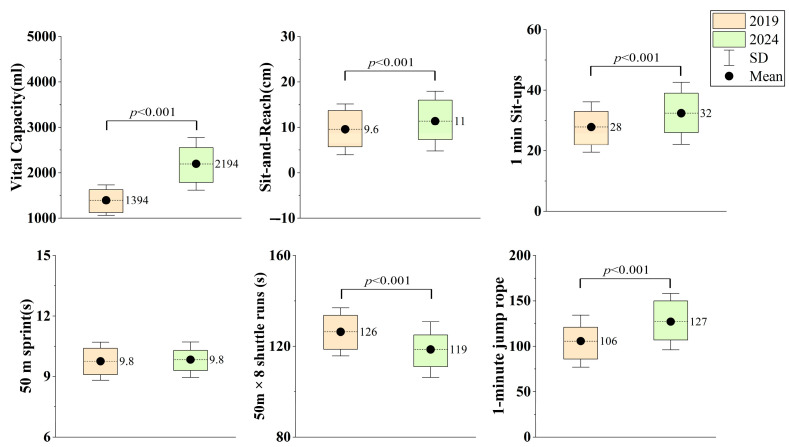
Comparison of physical fitness among girls in 2019 and 2024.

Among primary school girls, BMI also increased significantly from 2019 to 2024 (Table 2). Significant improvements were observed in vital capacity (t = −34.02, *p* < 0.001, d = 1.58), sit-up performance (t = −10.21, *p* < 0.001, d = 0.47), and sit-and-reach performance (t = −6.20, *p* < 0.001, d = 0.29). The completion time of the 50 m × 8 shuttle run decreased significantly, indicating improved speed–agility endurance performance (t = 14.29, *p* < 0.001, d = 0.66). Jump rope performance also increased significantly (t = −15.30, *p* < 0.001, d = 0.71). However, no significant difference was observed in 50 m sprint performance between 2019 and 2024 (t = −1.80, *p* = 0.072, d = 0.08) (Figure 3).

All significant differences in physical fitness indicators remained statistically significant after FDR correction.

The distribution of BMI categories was further examined. From 2019 to 2024, the proportion of overweight and obesity increased among boys, with a significant change in BMI category distribution (χ^2^ = 20.906, *p* < 0.001). In contrast, no significant change was observed among girls (χ^2^ = 3.949, *p* = 0.139) (Table 2).

### 3.3. Associations Between Dietary Behaviors and Physical Fitness Outcomes in Primary School Students

After adjustment for age, BMI, survey year, physical activity, and sleep duration, dietary behaviors showed limited associations with physical fitness indicators. Among boys, breakfast consumption was positively associated with one-minute jump rope performance (β = 0.058, *p* = 0.007, FDR-p = 0.020). Egg intake was positively associated with sit-up performance (β = 0.057, *p* = 0.024), although this association was attenuated after FDR correction. Milk consumption showed a positive association with jump rope performance (β = 0.043, *p* = 0.047), but did not remain significant after correction.

Among girls, egg intake was positively associated with one-minute sit-up performance (β = 0.058, *p* = 0.017, FDR-p = 0.051), while other dietary factors showed no significant associations after multiple comparison correction. Overall, breakfast, egg, and milk consumption demonstrated relatively weak independent associations with physical fitness outcomes after adjustment for potential confounders (Table 3).

Type III ANOVA was performed to examine the overall association between sugary beverage consumption categories and physical fitness indicators after adjusting for age, BMI, survey year, physical activity, sleep duration, breakfast consumption, egg intake, and milk intake (Table 4). Among boys, sugary beverage consumption categories showed a significant overall effect on 1 min sit-up performance (F = 2.141, *p* = 0.046), whereas no significant effects were observed for other fitness indicators. Among girls, significant overall effects of sugary beverage consumption categories were observed for 1 min sit-up performance (F = 3.930, *p* = 0.001), 50 m sprint performance (F = 4.620, *p* < 0.001), 50 m × 8 shuttle runs (F = 2.913, *p* = 0.008), and jump rope performance (F = 3.133, *p* = 0.005).

## 4. Discussion

In this study, physical fitness among 10–12-year-old students in Ningxia improved substantially from 2019 to 2024, with significant improvements observed in cardiorespiratory fitness, muscular endurance, flexibility, and speed-agility performance. Meanwhile, BMI increased during the same period, particularly among boys, indicating a coexistence of improved fitness and potential concerns regarding weight status. After adjustment for major confounders, dietary behaviors showed relatively modest associations with physical fitness outcomes, suggesting that changes in children’s fitness may reflect the combined influence of dietary patterns, lifestyle behaviors, and broader environmental factors.

Although physical fitness improved from 2019 to 2024, the concurrent increase in BMI warrants careful interpretation. Some increases in body mass may reflect normal growth and developmental changes during late childhood; however, the rising prevalence of overweight and obesity, particularly among boys, may indicate a potential risk of excessive weight gain and future metabolic consequences [34]. Therefore, continued monitoring of weight status and promoting healthy body composition development remain important, even in the context of improving physical fitness.

Dietary behaviors showed relatively modest associations with physical fitness outcomes after adjustment for survey year, age, BMI, physical activity, and sleep duration. Consumption of eggs and milk demonstrated positive associations with some fitness indicators, particularly muscular endurance-related outcomes; however, most associations were attenuated after correction for multiple comparisons. Similarly, sugary beverage consumption was associated with several fitness components, especially among girls. These findings suggest that dietary behaviors may contribute to variations in physical fitness during late childhood, although their independent effects appear to be relatively limited compared with broader lifestyle and environmental factors [35,36].

Although the observed associations between individual dietary behaviors and physical fitness were relatively modest, the potential biological relevance of protein-rich foods warrants consideration. Eggs and milk provide high-quality protein and essential micronutrients, including calcium and vitamins, which contribute to muscle and bone development during childhood [37,38,39,40]. Adequate protein intake supports muscle protein synthesis and may partly explain why these foods have been linked with physical performance in previous studies [41,42]. However, in the present study, the associations of egg and milk consumption with fitness outcomes were attenuated after adjustment for multiple comparisons, suggesting that the influence of individual dietary factors may be modest and likely interacts with other lifestyle factors, including physical activity and overall dietary patterns.

In contrast, sugary beverage consumption showed associations with several physical fitness indicators, particularly among girls. Higher consumption categories were associated with poorer performance in speed-agility related tests and jumping ability, suggesting a potential unfavorable relationship between excessive intake of sugar-sweetened beverages and physical performance during late childhood. Higher categories of sugary beverage consumption showed differences in several fitness indicators, particularly among girls.

Sugar-sweetened beverages provide substantial amounts of added sugars and energy with limited nutritional value, and excessive consumption has been linked to increased risks of weight gain and adverse metabolic profiles in children [23,43,44]. The high glycemic load associated with added sugars may further contribute to impaired metabolic regulation and increased obesity risk [44,45]. However, the cross-sectional nature of these associations prevents causal interpretation, and further longitudinal studies are needed to clarify the relationship between sugary beverage intake and physical fitness trajectories.

The observed improvements in cardiorespiratory fitness, muscular endurance, and speed-agility performance may reflect broader changes in children’s living environments and health promotion strategies in China. Recent initiatives emphasizing school-based physical education, extracurricular sports participation, and regular fitness monitoring may have contributed to increased opportunities for physical activity among primary school students. In addition, national policies such as the “Double Reduction” policy may have indirectly supported healthier daily routines by reducing academic-related burdens. However, as physical activity patterns and policy implementation were not directly measured in the present study, these factors should be considered potential explanations rather than confirmed determinants of the observed fitness improvements.

From a public health perspective, our findings highlight the importance of integrated strategies combining physical activity promotion, healthy dietary behaviors, and weight management during late childhood. Although dietary behaviors showed relatively modest independent associations with physical fitness, schools and families should continue to promote balanced dietary patterns, including adequate consumption of nutrient-dense foods such as eggs and dairy products, while reducing excessive intake of sugar-sweetened beverages. In addition, the increasing prevalence of overweight and obesity, particularly among boys, indicates the need for regular monitoring of weight status alongside physical fitness assessment. Given that lifestyle behaviors established during childhood often persist into later life, early school- and family-based health promotion strategies may provide long-term benefits for population health [46,47,48,49].

This study has several limitations. First, dietary assessment was limited to four selected behaviors and did not capture overall dietary quality or patterns. Second, although important demographic and lifestyle covariates were adjusted, socioeconomic status, family environment, and pubertal development were unavailable and may have contributed to residual confounding. Third, because school-level identifiers were unavailable, potential clustering effects related to the school-based sampling design could not be fully addressed. Finally, the repeated cross-sectional design limits causal inference and individual-level longitudinal interpretation. Future studies incorporating comprehensive dietary assessments, maturation indicators, and multilevel approaches are warranted.

## 5. Conclusions

In summary, children aged 10–12 years in Ningxia showed significant improvements in multiple physical fitness indicators between 2019 and 2024, accompanied by an increase in BMI and a shift toward higher BMI categories among boys. After adjustment for demographic and lifestyle factors, dietary behaviors showed relatively modest associations with physical fitness outcomes, with associations observed for several dietary factors, including sugary beverage consumption, particularly among girls. These findings suggest that recent changes in childhood physical fitness may reflect the combined influence of secular trends, lifestyle behaviors, and broader environmental factors. Comprehensive school-based strategies integrating physical activity promotion, healthy dietary behaviors, and weight monitoring may help support healthy development during late childhood.

## Figures and Tables

**Table 1:** Methods and criteria of the physical fitness test.

Component	Test Indicator	Description
Body composition	Height (cm) and weight (kg)	Height and weight were measured to assess body composition. Participants, barefoot and without heavy clothing or items, stood with their backs against a stadiometer. They maintained a posture with heels, sacrum, and scapulae in contact with the vertical surface, and their heads in a horizontal position. Measurements were recorded to the nearest 0.1 cm for height and 0.1 kg for weight.
Cardiorespiratory function	Vital capacity (mL)	For the test, subjects remained standing and were asked first to inhale as deeply as possible. They then sealed their lips firmly around the blowing nozzle and exhaled with maximum effort until no air remained. Two trials were performed, and the best score was used for subsequent analysis.
Speed	50 m sprint (s)	The 50 m sprint test is conducted with a standardized standing start. To ensure test validity, participants are grouped for testing, with each group comprising at least two athletes.
Flexibility	Sit and reach (cm)	During the seated forward bend test, the subject must remain seated with both legs fully extended and knees straight. Hands should be crossed, with one hand on top of the other (palms facing down). They slowly extended their hands as far as possible along the measuring scale. The maximum distance reached, measured in centimeters (cm), was recorded. Two trials were performed, and the highest score was used for analysis.
Core strength	1 min sit-up	The one-minute sit-up test required participants to perform abdominal curl-ups with their hands behind their heads and their knees flexed to 90 degrees. The total number of correctly executed repetitions completed within the time limit was recorded as the score.
Endurance	50 m × 8 shuttle run (s)	The 50 m × 8 shuttle run was administered to primary school students, and their completion times were recorded to the nearest 0.1 s.
Coordination	1 min rope-skipping test	Designed to evaluate lower-limb power, coordination, and endurance, the one-minute rope-skipping test required participants to perform as many valid rope-skipping jumps as possible within the allotted time, using an adjustable rope on a flat surface under timed conditions.

**Table 2:** Changes in BMI category distribution among primary school students from 2019 to 2024.

Sex	Year	Normal Weight n (%)	Overweight n (%)	Obesity n (%)	χ^2^	*p*
Boys	2019	676 (93.63%)	44 (6.09%)	2 (0.28%)	20.906	<0.001
	2024	1078 (87.08%)	151 (12.20%)	9 (0.73%)		
Girls	2019	645 (88.97%)	75 (10.34%)	5 (0.69%)	3.949	0.139
	2024	1164 (90.58%)	105 (8.17%)	16 (1.25%)		

**Table 3:** Associations of breakfast, egg, and milk consumption with physical fitness outcomes in primary school children: multivariable linear regression analysis.

Sex	Fitness Indicator	Breakfast β (*p*; FDR-p)	Egg β (*p*; FDR-p)	Milk β (*p*; FDR-p)
Boys	Vital Capacity	0.013 (0.517; 0.651)	0.010 (0.651; 0.651)	0.032 (0.120; 0.360)
	Sit-and-Reach	0.018 (0.483; 0.483)	0.041 (0.112; 0.337)	−0.029 (0.246; 0.369)
	1 min Sit-up	0.028 (0.247; 0.247)	0.057 (0.024; 0.072)	0.032 (0.190; 0.247)
	50 m Sprint	−0.011 (0.653; 0.653)	0.018 (0.470; 0.653)	−0.053 (0.032; 0.097)
	50 m × 8 shuttle runs	−0.032 (0.183; 0.183)	0.057 (0.021; 0.063)	−0.042 (0.085; 0.127)
	1 min jump rope	0.058 (0.007; 0.020)	0.014 (0.519; 0.519)	0.043 (0.047; 0.071)
Girls	Vital Capacity	−0.001 (0.962; 0.967)	−0.001 (0.967; 0.967)	0.032 (0.095; 0.283)
	Sit-and-Reach	0.039 (0.094; 0.282)	0.029 (0.240; 0.359)	0.004 (0.885; 0.885)
	1 min Sit-up	0.015 (0.505; 0.505)	0.058 (0.017; 0.051)	0.023 (0.338; 0.505)
	50 m Sprint	0.001 (0.961; 0.961)	−0.040 (0.108; 0.325)	−0.016 (0.520; 0.779)
	50 m × 8 shuttle runs	−0.034 (0.129; 0.129)	−0.037 (0.116; 0.129)	0.053 (0.026; 0.077)
	1 min jump rope	0.035 (0.085; 0.128)	0.031 (0.163; 0.163)	0.038 (0.084; 0.128)

β represents standardized regression coefficients. Models were adjusted for age, BMI, survey year, physical activity, and sleep duration. FDR-p values were calculated using the Benjamini–Hochberg procedure.

**Table 4:** Differences between sugary beverage consumption and physical fitness indicators based on Type III ANOVA.

Sex	Fitness Indicator	F Value	*df*	*p* Value
Boys	Vital Capacity	1.200	6	0.303
	Sit-and-Reach	2.016	6	0.060
	1 min Sit-up	2.141	6	0.046
	50 m Sprint	1.477	6	0.182
	50 m × 8 shuttle runs	0.676	6	0.669
	1 min jump rope	1.795	6	0.096
Girls	Vital Capacity	1.059	6	0.385
	Sit-and-Reach	1.946	6	0.070
	1 min Sit-up	3.930	6	0.001
	50 m Sprint	4.620	6	<0.001
	50 m × 8 shuttle runs	2.913	6	0.008
	1 min jump rope	3.133	6	0.005

## Data Availability

The data presented in this study are available upon request from the corresponding author. The data are not publicly available due to confidentiality reasons.

## Abbreviations

The following abbreviations are used in this manuscript:

CNSSCH	Chinese National Survey on Students’ Constitution and Health
BMI	Body Mass Index
MVPA	Moderate to Vigorous Physical Activity
